# Channel Estimation for RIS-Assisted MIMO Systems in Millimeter Wave Communications

**DOI:** 10.3390/s23125516

**Published:** 2023-06-12

**Authors:** Ying Liu, Honggui Deng, Chengzuo Peng

**Affiliations:** School of Physics and Electronics, Central South University, Lushan South Road, Changsha 410083, China; 212212057@csu.edu.cn (Y.L.); 202212087@csu.edu.cn (C.P.)

**Keywords:** reconfigurable intelligent surface, LMMSE, channel estimation, mmWave

## Abstract

The large number of estimated parameters in a reconfigurable intelligent surface (RIS) makes it difficult to achieve accurate channel estimation accuracy in 6G. Therefore, we suggest a novel two-phase channel estimation framework for uplink multiuser communication. In this context, we propose an orthogonal matching pursuit (OMP)-based linear minimum mean square error (LMMSE) channel estimation approach. The OMP algorithm is used in the proposed algorithm to update the support set and pick the columns of the sensing matrix that are most correlated with the residual signal, which successfully reduces pilot overhead by removing redundancy. Here, we use LMMSE’s advantages for handling noise to address the problem of inadequate channel estimation accuracy when the signal-to-noise ratio (SNR) is low. Simulation findings demonstrate that the proposed approach outperforms least-squares (LS), traditional OMP, and other OMP-based algorithms in terms of estimate accuracy.

## 1. Introduction

One of the important 6G technologies is reconfigurable intelligent surface (RIS), which has received a lot of research attention. It consists of a controller and a sizable number of inexpensive passive reflecting components without RF chains. The wireless communication environment can be intelligently manipulated by RIS by adjusting the coefficients of its constituents [1,2,3]. Wireless communication coverage, throughput, and energy efficiency can all be considerably increased by coherently combining and guiding wireless signals in the desired direction [4,5]. Due to passive reflection, RIS also consumes less energy and has lower hardware costs than conventional active relays or beamforming methods [6,7]. However, precise channel state information (CSI) is needed for RIS to operate to its full potential. Therefore, obtaining precise CSI is essential for RIS-assisted communication systems.

To get accurate CSI, there are two key obstacles to overcome. First, many passive elements are incapable of actively transmitting or receiving signals because they lack the ability to analyze signals. Only active antennas on the base station (BS) and user equipment (UE) can be used to determine CSI [8,9]. As a result, it is difficult to estimate BS-RIS and RIS-UE channels using RIS-assisted channel estimation. Second, a large number of antennas for multiple input multiple output (MIMO) systems leads to decreased channel estimate accuracy [10]. Therefore, channel estimation is difficult in RIS-aided MIMO communication systems.

Many different approaches have recently been put forth to investigate channel estimation in RIS-assisted communication systems [11,12,13,14,15,16,17,18]. The work [11] specifically proposed an ON/OFF-based channel-estimating approach to directly estimate cascaded channels. In accordance with this technique, *N* time slots are sufficient to accurately estimate all reflective channels of the user in the scenario that there is no received noise at the BS, thus decreasing the pilot overhead necessary for channel estimation. However, channel estimation accuracy may be decreased since only one RIS element can reflect the pilot signal to the BS at a time slot. The work [12] proposed a channel estimation strategy based on discrete Fourier transform (DFT), which can still perfectly estimate all cascaded channels of a user through *N* time slots. In addition, since all RIS components remain turned on in each time slot, it can achieve higher estimation accuracy than the ON/OFF strategy, but the pilot overhead is still high. The work [13] proposed a channel estimation method based on row-structured sparsity utilizing the traditional orthogonal matching pursuit (OMP) algorithm in compressed sensing (CS) for channel estimation. The work [14] developed a unique angle domain cascaded channel sparsity-based channel estimation technique based on conventional CS. By leveraging the double-structured sparsity of the angle cascaded channels, the work [15] developed a double-structured orthogonal matching pursuit (DS-OMP) based cascaded channel estimation scheme. Compared with the prior method, this one requires less pilot overhead for channel estimate. Under low signal-to-noise ratios (SNR), the works [13,14,15] were unable to obtain satisfactory estimation accuracy. The above papers only use incomplete sparsity, which can lead to redundant pilot consumption. Taking advantage of the specific triple structure sparsity of cascaded channels, [16] proposed a multi-user joint estimation algorithm that significantly reduces pilot overhead. However, this algorithm introduced a hypothetical condition that is difficult to achieve in practical situations. The work [17] proposed a multidimensional orthogonal matching pursuit strategy for compressive channel estimation, computing the projections on a set of independent dictionaries instead of a single large dictionary to achieve high-accuracy channel estimation at reduced complexity. However, multiple independent dictionaries bring additional pilot overhead. By utilizing the common sparsity between different subcarriers and the dual structure sparsity of the angle cascaded channel matrix, the work [18] proposed a data-driven cascaded channel estimation method to accurately detect channel support using denoising neural networks. This work introduced deep learning to solve the denoising problem of channel estimation which inevitably led to increased complexity.

According to the discussion above, there is still much opportunity for improvement in the situation of low SNR in order to achieve improved channel estimation accuracy with less pilot overhead, which is also essential in real applications. In the case of low SNR, the channel is affected by various non-ideal factors such as noise. This leads to an increase in channel estimation error. It is necessary to model non-ideal channels and develop adaptive algorithms to counteract their effects. Consequently, we propose an algorithm that can utilize the noise processing advantages of LMMSE on the basis of the OMP algorithm to reduce channel estimation errors under low SNR. We emphasize our significant contributions in the following summary of the paper:

- We present a linear minimal mean square error (LMMSE) channel estimation approach based on OMP to estimate the BS-UE direct channel without taking RIS into account in the model we develop in mmWave MIMO communication system. We employ the OMP approach to obtain the support set and the LMMSE algorithm to estimate the channel after converting the channel estimation problem into a sparse signal recovery problem. The proposed approach can increase estimation accuracy while requiring less pilot overhead.
- By using the double-structured sparsity of the angle cascaded channel in mmWave, we present an LMMSE channel estimation technique based on DS-OMP to estimate the BS-RIS-UE cascaded channel. The DS-OMP approach is used to get the support sets for the angle cascaded channel, and the LMMSE algorithm is used to estimate the channel. The proposed technique successfully manages noise to produce a more accurate estimation.

The rest of this article is arranged as follows. The system model is introduced in Section 2, which also discusses the direct channel and cascade channel estimation problems. We present the proposed channel estimate algorithms for direct and cascaded channels, respectively, in Section 3. The simulation results in Section 4 demonstrate the viability of the proposed approach. Section 5 presents the conclusion of the article.

## 2. System Model and Problem Formulation

In this section, we introduce the system models of the two stages of channel estimation and describe their channel estimation issues, respectively.

### 2.1. Direct Channel

**Figure 1:**
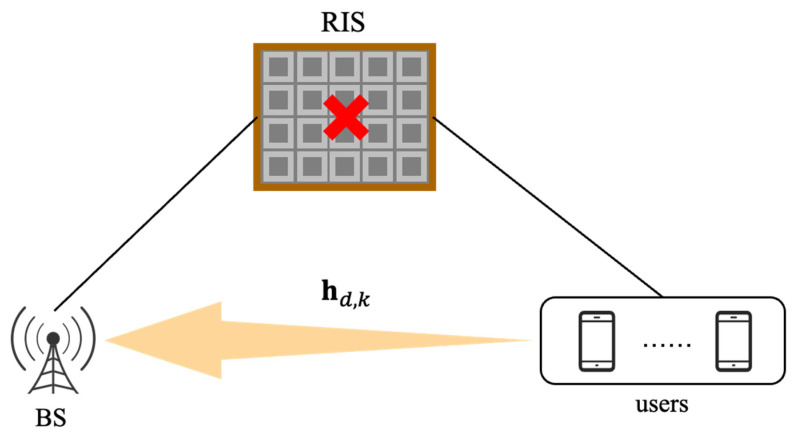
Multi-user mmWave MIMO system without considering RIS.

As shown in Figure 1, we take into account a wireless system with K single-antenna users interacting with a base station (BS) in the uplink while the BS has M antennas and uses a uniform planar array (UPA) [19]. All RIS components remain closed when there is communication between the UE and the BS. $h_{d,k}$, k=1,2,⋯,K, denotes the direct channel from the kth user to the BS. In this paper, we simulate the direct channel using the Saleh-Valenzuela channel [20,21] as

(1) $$h_{d,k}=\sqrt{\frac{M}{L_{d,k}}}\sum_{l_{1}=1}^{L_{d,k}}\alpha_{l_{1}}^{d,k}b\left(\vartheta_{l_{1}}^{d,k},\psi_{l_{1}}^{d,k}\right),\;\forall k,$$

where $L_{d,k}$ is the number of paths between the BS and UE, $\alpha_{l_{1}}^{d,k}$, $\vartheta_{l_{1}}^{d,k}(\psi_{l_{1}}^{d,k})$ denotes the complex gain and the azimuth (elevation) angle at the BS for the $l_{1}$ path, respectively. $b\left(\vartheta_{l_{1}}^{d,k},\psi_{l_{1}}^{d,k}\right)$ represents the normalized array steering vector at the BS. $b\left(\vartheta_{l_{1}}^{d,k},\psi_{l_{1}}^{d,k}\right)$ can be represented by

(2) $$b\left(\vartheta_{l_{1}}^{d,k},\psi_{l_{1}}^{d,k}\right)={\left[1,e^{j2\pi dsin\vartheta sin\psi /\lambda },\cdots ,e^{j2\pi (M_{1}-1)dsin\vartheta sin\psi /\lambda }\right]}^{T}\;⨂{\left[1,e^{j2\pi dcos\psi /\lambda },\cdots ,e^{j2\pi (M_{2}-1)dcos\psi /\lambda }\right]}^{T},$$

where d is the antenna spacing, λ is the signal wavelength, and ⨂ represents the Kronecker product. We define the array response matrix as $A_{R}=[\alpha_{1}^{d,k}\left(\vartheta_{1}^{d,k},\psi_{1}^{d,k}\right),\alpha_{2}^{d,k}\left(\vartheta_{2}^{d,k},\psi_{2}^{d,k}\right),\cdots ,\alpha_{L_{d,k}}^{d,k}\left(\vartheta_{L_{d,k}}^{d,k},\psi_{L_{d,k}}^{d,k}\right)]$, which constitutes the dictionary of our CS formulation, and the rows of the matrix are orthogonal [22]. Then, we can rewrite (1) utilizing the array response matrix, $A_{R}$, as follows:

(3) $$h_{d,k}=A_{R}diag(\alpha ),$$

where $\alpha =[\alpha_{1}^{d,k},\alpha_{2}^{d,k},\cdots ,\alpha_{L_{d,k}}^{d,k}]$. All users transmit the known pilot symbols to the BS over Q time slots in accordance with the commonly utilized orthogonal pilot transmission scheme [23] for uplink channel estimation. At this point, all RIS components are turned off; i.e.,

(4) $$\phi_{n,q}=0,\;n=1,2,\cdots ,N,q=1,2,\cdots ,Q.$$

Specifically, in the qth (q=1,2,···,Q) time slot, the effective received signal for the kth user at the BS is denoted by the following equation:

(5) $$y_{k,q}=h_{d,k}x_{k,q}+n_{k,q},$$

where $x_{k,q}$ is the pilot symbol sent by the kth user, and $n_{k,q}\sim CN\left(0,\sigma^{2}I_{M}\right)$ is the M×1 received additive white Gaussian noise (AWGN), with $\sigma^{2}$ representing the noise power.

Vectorizing the received signal, $y_{k,q},$ is fundamental to formulate the channel estimation issue as

(6) $$y_{k,q}=A_{R}diag\left(\alpha \right)x_{k,q}+n_{k,q},$$

### 2.2. Cascaded Channel

**Figure 2:**
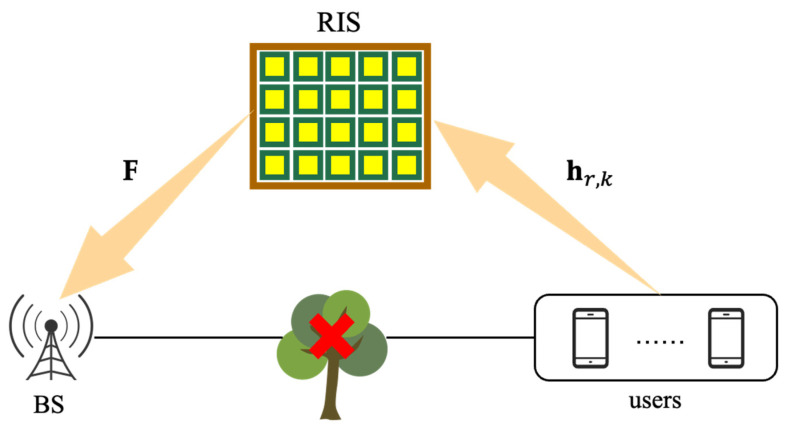
RIS-aided multi-user mmWave MIMO system.

As shown in Figure 2, we keep all the RIS elements turned on. There are M antennas and N elements in BS and RIS, respectively, and they are both UPA to serve K single-antenna users simultaneously. $F\in C^{N\times M}$ indicates the RIS-BS channel; $h_{r,k}\in C^{N\times 1}$ indicates the channel from the kth user to the RIS (k=1,2,⋯,K). To obtain the channel between RIS and BS, we employ the Saleh-Valenzuela channel model, as shown below:

(7) $$F=\sqrt{\frac{NM}{L_{F}}}\sum_{l_{2}=1}^{L_{F}}\alpha_{l_{2}}^{F}b(\vartheta_{l_{2}}^{F_{r}},\psi_{l_{2}}^{F_{r}})a{\left(\vartheta_{l_{2}}^{F_{t}},\psi_{l_{2}}^{F_{t}}\right)}^{T},$$

where $L_{F}$ is the quantity of paths connecting the RIS and BS, and $\alpha_{l_{2}}^{F}$, $\vartheta_{l_{2}}^{F_{r}}(\psi_{l_{2}}^{F_{r}})$, $\vartheta_{l_{2}}^{F_{t}}(\psi_{l_{2}}^{F_{t}})$ is the complex gain, the azimuth (elevation) angle at the BS and RIS for the $l_{2}$ path, respectively. Likewise, the channel between UE and RIS can be depicted by

(8) $$h_{r,k}=\sqrt{\frac{N}{L_{r,k}}}\sum_{l_{3}=1}^{L_{r,k}}\alpha_{l_{3}}^{r,k}a(\vartheta_{l_{3}}^{r,k},\psi_{l_{3}}^{r,k}),$$

where $L_{r,k}$ is the quantity of paths between the kth user and RIS, $\alpha_{l_{3}}^{r,k}$, $\vartheta_{l_{3}}^{r,k}(\psi_{l_{3}}^{r,k})$, is the complex gain, the azimuth (elevation) angle at the RIS for the $l_{3}$ path, respectively. $a\left(\vartheta ,\psi \right)\in C^{N\times 1}$ represents the steering vector for the normalized array at the RIS. For a typical $N_{1}\times N_{2}$(${N=N}_{1}\times N_{2}$) UPA, $a\left(\vartheta ,\psi \right)$ can be defined by [24]

(9) $$a(\vartheta ,\psi )=\frac{1}{\sqrt{N}}[e^{-j2\pi dsin(\vartheta )cos(\psi )n_{1}/\lambda }]⨂[e^{-j2\pi dsin(\psi )n_{2}/\lambda }],$$

where $n_{1}=[0,1,\cdots ,N_{1}-1]$, $n_{2}=[0,1,\cdots ,N_{2}-1]$, d is the spacing between the antennas, λ is the wavelength, and ⨂ represents the Kronecker product. We define the kth user’s N×M cascaded channel as $H_{k}=Fdiag(h_{r,k})$, and we convert it to angular domain representation as

(10) $$H_{k}=U_{M}{\tilde{H}}_{k}U_{N}^{T},$$

where ${\tilde{H}}_{k}$ is the N×M angle cascaded channel, and $U_{M}$ and $U_{N}$ are the BS’s and RIS’s respective M×M and N×N dictionary unitary matrices [20]. There are a few non-zero elements in the angle cascaded channel, which exhibits sparsity, as a result of the minimal scattering near BS and RIS.

All users transmit known pilot symbols to BS through RIS in Q time periods using an orthogonal pilot transmission approach to estimate the uplink channel. The effective received signal for the *k*th user at the BS in the qth (q=1,2,···,Q) time slot can be expressed as $y_{k,q}\in C^{M\times 1}$ after the direct channel effect between BS and UE has been removed as

(11) $$y_{k,q}=Fdiag\left(h_{r,k}\right)\theta_{q}x_{k,q}+n_{k,q},$$

where $x_{k,q}$ is the pilot symbol that the kth user sends, $\theta_{q}={\left[\theta_{q,1},\theta_{q,2},\cdots ,\theta_{q,N}\right]}^{T}$ is the reflecting vector of RIS, the reflecting coefficient at the nth RIS element (n=1,2,⋯,N) in the qth time slot is given by $\theta_{q,n}$, and $n_{k,q}\sim CN\left(0,\sigma^{2}I_{M}\right)$ is the M×1 received noise with $\sigma^{2}$ representing the noise power. According to $H_{k}=Fdiag(h_{r,k})$, (11) can be written as

(12) $$y_{k,q}={H_{k}\theta }_{q}x_{k,q}+n_{k,q}.$$

For Q time slots after pilot transmission, let $x_{k,q}=1$, and we obtain the overall measurement matrix of $Y_{k}=[y_{k,1},y_{k,2},\cdots ,y_{k,Q}]$ as

(13) $$Y_{k}=H_{k}\Theta +N_{k},$$

where $\Theta =[\theta_{1},\theta_{2},\cdots ,\theta_{Q}]$, and $N_{k}=\left[n_{k,1},n_{k,2},{\cdots ,n}_{k,Q}\right]$. We can obtain the transformation of $Y_{k}$ by substituting (10) into (13) as

(14) $$Y_{k}=U_{M}{\tilde{H}}_{k}U_{N}^{T}\Theta +N_{k}.$$

${\tilde{Y}}_{k}={\left(U_{M}^{H}Y_{k}\right)}^{H}$ is the effective measurement matrix, and ${\tilde{N}}_{k}={\left(U_{M}^{H}N_{k}\right)}^{H}is\;the$ effective noise matrix. Based on the above formula, we can determine a CS model as follows

(15) $${\tilde{Y}}_{k}=\tilde{\Theta }{\tilde{H}}_{k}^{H}+{\tilde{N}}_{k},$$

where $\tilde{\Theta }={\left(U_{N}^{T}\Theta \right)}^{H}$ denotes the sensing matrix. By making full use of the double structured sparsity of the angle cascaded channel, we can estimate the channel. However, under the premise of low SNR, the estimation accuracy of channel estimation algorithms based on DS-OMP still needs to be improved.

## 3. Proposed Channel Estimation Scheme

### 3.1. Direct Channel Estimation

Based on (5), the channel between BS and UE for the kth user can be estimated separately. Traditional channel estimation algorithms, such as the LS algorithm, have obvious advantages due to their simple implementation and low computational complexity. However, due to the lack of noise processing, the estimation accuracy cannot meet the requirements of practical applications. In addition, its estimation performance is very limited in certain specific scenarios. Based on this, we propose an OMP-based LMMSE algorithm, which can effectively solve the challenges above. Finally, we evaluate the proposed algorithm’s computational complexity.

#### 3.1.1. Least Square (LS) Algorithm

The goal of the LS algorithm is to reduce the distance between signals that are received from their optimal distance; i.e.,

(16) $${\hat{H}}_{LS}=arg\;min(Y-{\hat{H}}_{LS}X){\left(Y-{\hat{H}}_{LS}\right)}^{H},$$

To minimize the sum of squares of errors, we make (16)’s first order partial derivative in relation to $\hat{H}$ equal to 0; i.e.,

(17) $${\hat{H}}_{LS}={\left(X^{H}X\right)}^{-1}X^{H}Y,$$

At this point, the sum of squares of the obtained estimates is the minimum, which is the solution of the LS channel estimation. The LS algorithm is frequently used in practice, has a low computing complexity, and is reasonably easy to implement. However, it has two significant issues that must be resolved. First, because the LS algorithm ignores the impact of noise, estimation accuracy is significantly impacted in low SNR situations. Second, the number of antennas at the BS is typically enormous, which could lead to overly large dimensionality of channel estimation problems, making it challenging to employ the LS algorithm for mmWave communication [24].

#### 3.1.2. Proposed LMMSE Algorithm Based on OMP

Firstly, to address the issue of noise processing in the LS algorithm, a weighting matrix, W, is added to the LS algorithm:

(18) $${\hat{H}}_{MMSE}=W{\hat{H}}_{LS}=R_{{H\hat{H}}_{LS}}{R_{{{\hat{H}}_{LS}\hat{H}}_{LS}}^{-1}\hat{H}}_{LS}=R_{{H\hat{H}}_{LS}}{\left(R_{HH}+\frac{\sigma_{Z}^{2}}{\sigma_{X}^{2}}I\right)}^{-1}{\hat{H}}_{LS}.$$

This is the minimum mean square error (MMSE) algorithm [25,26]. However, the matrix inversion requires a large amount of computation and continuous recalculation, which greatly occupies computational resources and has poor real-time performance. Consequently, the MMSE algorithm has limitations in practical applications. The LMMSE algorithm performs linear smoothing on the basis of the MMSE algorithm, considering the MMSE algorithm’ need to compute ${\left(R_{HH}+\frac{\sigma_{Z}^{2}}{\sigma_{X}^{2}}\right)}^{-1}$; as the noise changes and the input signal changes, the inversion operation becomes very complex, so, the LMMSE algorithm uses expectation to displace the $\frac{\sigma_{Z}^{2}}{\sigma_{X}^{2}}$. If we let $SNR=\frac{E(X^{2})}{\sigma^{2}}$, $\beta =\frac{E(X^{2})}{E({1/X}^{2})}$, the estimated channel is obtained from the LMMSE algorithm as [27]

(19) $${\hat{H}}_{LMMSE}=R_{{h_{d,k}\hat{H}}_{LS}}{\left(R_{h_{d,k}h_{d,k}}+\frac{\beta }{SNR}\right)}^{-1}{\hat{H}}_{LS},$$

where $R_{h_{d,k}{\hat{H}}_{LS}}$ is the cross-correlation matrix of ${\hat{H}}_{LS}$; $h_{d,k}$, $R_{h_{d,k}h_{d,k}}$ denotes the autocorrelation matrix of $h_{d,k}$; and β is a channel-modulation-type parameter that in this case, we set as β=1. We assume that the number of propagation paths between the channels BS and UE is $L_{d,k}=8$ and that the formula additionally contains a matrix inversion operation. In other words, we must do an 8 by 8 matrix inversion.

Then, as the number of entries to be estimated in the CS method is proportional to the sparsity, and its sparsity level is considerably lower than MK, we utilize the CS algorithm to overcome the difficulties of implementing the LS algorithm in mmWave communication. For direct channel estimation, we take into account a conventional OMP approach and combine it with the LMMSE algorithm, which has additional advantages in noise processing. Algorithm 1 provides a summary of our scheme.
**Algorithm 1** Proposed LMMSE Algorithm Based on OMP**Input:** Receive signal, ${\tilde{Y}}_{k}$; sensing matrix, $\tilde{\Theta }$; true channel matrix, $h_{d,k}$; a scalar parameter for LMMSE estimation, β; SNR; BS’s number of antennas, M; the number of users, K; and the number of paths (BS-UE), $L_{d,k}$.**Initialization:** ${\hat{h}}_{d,k}$ $=0_{M\times K}$, $R_{HH}=0_{M\times K}$, $R_{HS}=0_{M\times K}$.1. for k=1,2,⋯,K do2.    $Y_{K}$ = ${\tilde{Y}}_{k}$(: , : , k), $R=Y_{K}$.3.    for $i=1,2,\cdots ,L_{d,k}$ do4.      Compute the term = ${\left|\tilde{\Theta }\prime R\right|}^{2}$.5.      Find the index of the maximum value in the term.6.      Find the column and row indices of the selected index.7.      for j=1,2,⋯,length(column) do8.          Find the corresponding rows in ${\tilde{\Theta }}_{i}$ and $Y_{K}$9.          Compute the LS of the channel for the selected column10.     end for11.   end for12.   $R_{HH}=h_{d,k}(:,k)h_{d,k}(:,k)$’; $R_{HH}=h_{d,k}(:,k){\hat{h}}_{OMP}$’.13.   Calculate the estimated channel matrix, ${\hat{h}}_{d,k},$ according to Equation (19).14. end for**Output:** Estimated channel matrix, ${\hat{h}}_{d,k}$.

The following is the explanation of Algorithm 1’s primary procedure. First, for each user, k, find the position of the largest element in the term in step 5. For each group column, perform steps 6–8 and save the column vector of the corresponding position in the sensing matrix to ${\tilde{\Theta }}_{i}$. We use Moore Penrose pseudo inverse to calculate ${\hat{h}}_{OMP}$ of the column j in step 9. Finally, ${\hat{h}}_{d,k}$ is obtained using the LMMSE algorithm based on (19) in step 13.

#### 3.1.3. Computational Complexity Analysis

Here, we examine the computational complexity of the three-layer loop that corresponds to the appropriate section of our OMP-based LMMSE algorithm’s major computational work. Specifically, for each user, k, L cycles are executed, and the complexity of each cycle is $O\left(KM\right)$. To calculate $\tilde{\Theta }\prime R$, we need to find the maximum value and calculate the estimated channel, ${\hat{h}}_{OMP}$. Therefore, the complexity from steps 1 to 12 is $O\left(KML^{2}\right)$. In step 13, we calculate matrix multiplication according to (19), where $R_{HS}$ and ${\hat{h}}_{OMP}$ are M×K matrix, and $R_{HH}$ is M×M matrix. Consequently, it is necessary to calculate $O\left(KM^{2}\right)$ times multiplication and addition. At the same time, using the pinv function to calculate the inverse matrix requires calculating $O\left(M^{3}\right)$ times multiplication and addition. Therefore, the complexity of step 13 is $O\left(KM^{3}\right)$. In summary, the computational complexity of our proposed algorithm is $O\left(KML^{2}+KM^{3}\right)$.

### 3.2. Cascaded Channel Estimation

Based on (15), for each user, k, we are able to estimate the angle cascaded channel. The traditional CS algorithm generates high pilot overhead while ensuring great estimation accuracy. Although the DS-OMP algorithm proposed in [15] reduces the pilot overhead of cascaded channel estimation to some extent, the estimation accuracy still needs to be improved in low SNR. Therefore, we propose an improved DS-OMP algorithm to achieve higher estimation accuracy in low SNR scenarios. Finally, the computational complexity of this algorithm is analyzed.

#### 3.2.1. Proposed LMMSE Algorithm Based on OMP

The matrix ${\tilde{H}}_{k}$ in (10) can be depicted using the following equation

(20) $${\tilde{H}}_{k}=\sqrt{\frac{MN}{L_{1}L_{2}}}\sum_{l_{2}}^{L_{1}}\sum_{l_{3}}^{L_{2}}\alpha_{l_{2}}^{F}\alpha_{l_{3}}^{r,k}\tilde{b}(\vartheta_{l_{2}}^{F_{r}},\psi_{l_{2}}^{F_{r}}){\tilde{a}}^{T}(\vartheta_{l_{2}}^{F_{t}}+\vartheta_{l_{3}}^{r,k},\psi_{l_{2}}^{F_{t}}+\psi_{l_{3}}^{r,k}),$$

where $\tilde{b}\left(\vartheta ,\psi \right)=U_{M}^{H}b(\vartheta ,\psi )$ and $\tilde{a}\left(\vartheta ,\psi \right)=U_{N}^{H}a(\vartheta ,\psi )$. Depending on the array steering vector in the $\left(\vartheta ,\psi \right)$ direction of $U_{N}$ and $U_{M}$, each one of them has just one non-zero element. In [15], a thorough discussion of the double-structured sparsity of the angular domain cascaded channel is presented.

**Figure 3:**
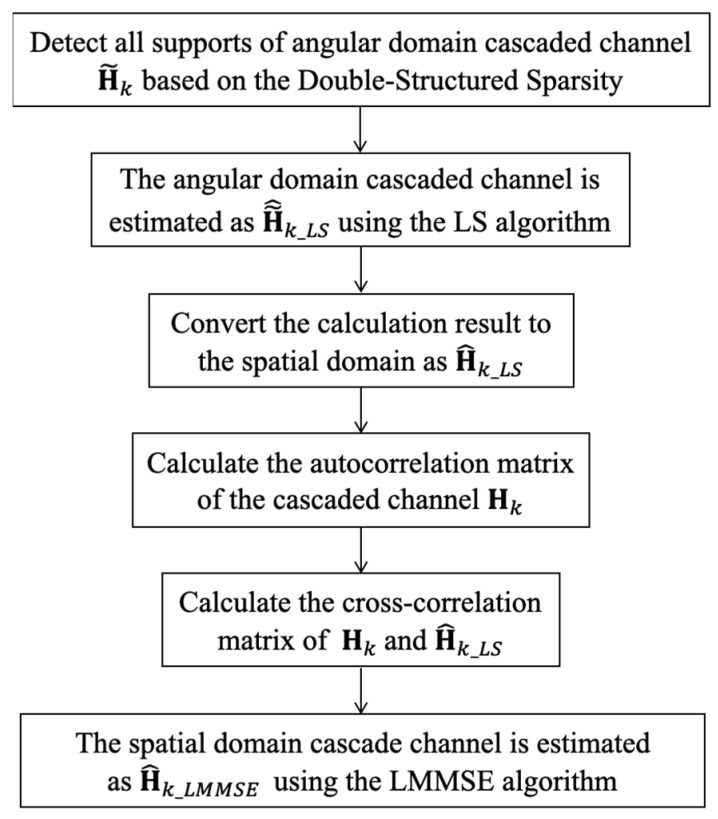
Proposed LMMSE algorithm based on OMP.

In this section, we consider integrating LMMSE into the classic OMP algorithm and propose an improved DS-OMP cascaded channel estimation scheme. The algorithm process is summarized in Figure 3.

The following explanation outlines the essential steps of the proposed method. First, by utilizing the sparsity of the double structure, we estimate the angle cascaded channel ${\tilde{H}}_{k}$’s completely public row support, partially public column support, and specific column support for k [15]. Once all supports have been identified, we use the LS algorithm to obtain the estimation matrix, ${\hat{\tilde{H}}}_{k\_LS}$**,** for the angle cascaded channel, ${\left\{{\hat{\tilde{H}}}_{k\_LS}\right\}}_{k=1}^{K}$, then use ${\hat{H}}_{k}=U_{M}^{H}{\hat{\tilde{H}}}_{k}U_{N}$ to transform ${\hat{\tilde{H}}}_{k\_LS}$ into the spatial cascaded channel ${\hat{H}}_{k\_LS}$. Next, we calculate the real cascaded channel $H_{k}$’s autocorrelation matrix of and the cross-correlation matrix of ${\hat{H}}_{k\_LS}$ and $H_{k}$. Then, the estimated cascaded channel matrix, ${\hat{H}}_{k\_LMMSE}$, is obtained using the following formula:

(21) $${\hat{H}}_{k\_LMMSE}=R_{H_{k}{\hat{H}}_{k\_LS}}{\left(R_{H_{k}H_{k}}+\frac{\beta }{SNR}\right)}^{-1}{\hat{H}}_{k\_LS},$$

where $R_{H_{k}{\hat{H}}_{k\_LS}}$ is the cross-correlation matrix of ${\hat{H}}_{k\_LS}$ and $H_{k}$, $R_{H_{k}H_{k}}$ is the autocorrelation matrix of $H_{k}$. We set β=1 here.

#### 3.2.2. Computational Complexity Analysis

The computational complexity of the proposed LMMSE scheme based on DS-OMP is examined in this section. The complexity of this algorithm mainly comes from three parts: completely common row support detection, partially common column support and specific column support detection, and channel estimation matrix calculation. The complexity of the first part mainly comes from energy calculation, with a complexity of $O\left(KM\right)$. The second part contains a triple loop, where the first loop has a count of $L_{1}$. The second cycle is K, and the third cycle is $L_{2}+{L_{c}}^{2}$, so the computational complexity of the second part is $O(KL_{1}(L_{2}+{L_{c}}^{2}))$. The third part involves matrix multiplication and inversion operations, and its complexity is $O(KN^{3})$. In summary, the computational complexity of the entire algorithm is $O\left(KM+KL_{1}\left(L_{2}+{L_{c}}^{2}\right)+KN^{3}\right)$.

## 4. Stimulation Results

To demonstrate the effectiveness of the proposed approach, we give the simulation results for the direct channel (BS-UE) and cascade channel (BS-RIS-UE) estimate phases in this section.

### 4.1. Direct Channel Estimation

The following simulation parameters are chosen: $M=64,\;K=8,\;L_{d,k}=8$, and $d_{BU}=$100 m (the distance between BS and UE). A total of 500 Monte Carlo simulations are performed using MATLAB R2019a in the simulation. For performance evaluation, we employ the normalized mean square error (NMSE), which is defined as

(22) $$NMSE=E\left[{\left\|{\hat{h}}_{d,k}-h_{d,k}\right\|}_{2}^{2}/{\left\|h_{d,k}\right\|}_{2}^{2}\right],$$

In the simulations that followed, we compared the NMSE performance of the proposed scheme with that of the LS algorithm, the conventional OMP method, and an upgraded OMP algorithm for channel estimation in order to demonstrate the proposed algorithm’s superiority.

**Figure 4:**
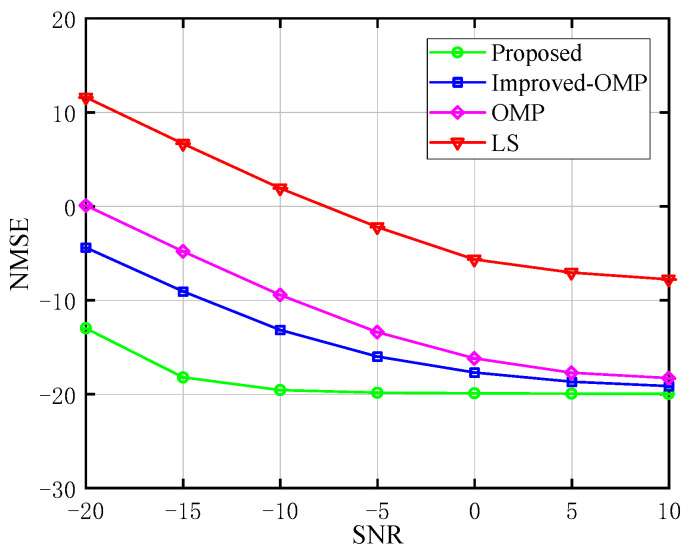
Performance evaluation of the NMSE for the BS-UE channels (compared with LS [25], conventional OMP algorithm [14], and improved OMP algorithm [26]).

The link between NMSE and SNR for the BS-UE channel estimation is shown in Figure 4. We contrast the proposed channel estimation algorithm’s NMSE with those of the LS, conventional, and improved OMP algorithms. The LS method is the least computationally complex of them all, but it ignores the effects of noise. The other two algorithms consider noise, but they perform poorly when the SNR is low. Our proposed channel estimation algorithm achieves higher estimation accuracy with acceptable computational complexity at low SNR. Since the LMMSE algorithm is a statistical estimation method aimed at minimizing the mean square error of channel estimation. It is based on the linear relationship between the received signal and the known transmitted signal sequence, while considering the channel noise and the correlation of the signal. In low SNR conditions, the noise in the received signal becomes the main interference factor and significantly affects the accuracy of channel estimation. The LMMSE algorithm can mitigate the impact of noise on channel estimation by optimizing the estimated mean square error. It achieves this by fully utilizing the known transmitted signal sequence and the linear relationship with the received signal. Consequently, the proposed scheme converges quickly to a performance platform. When SNR=−20 dB, the proposed algorithm can achieve an estimation accuracy of about $10^{-1.3}$ orders of magnitude, which has significant advantages compared with the three algorithms considered. When SNR=10 dB, about $10^{-2}$ orders of magnitude estimation accuracy can be attained with the proposed scheme, and it is superior to the other three algorithms. Therefore, we can see the superiority of the proposed algorithm.

### 4.2. Cascaded Channel Estimation

The following simulation parameters are chosen: $M=64,\;N=256,\;K=8,\;L_{F}=3$, $L_{r,k}=8$, $L_{c}=0,4,6$ (the number of common paths for ${\left\{h_{r,k}\right\}}_{k=1}^{K}$), $d_{RU}=$10 m (the distance between RIS and UE), $d_{BU}=$100 m. A total of 500 Monte Carlo simulations are performed using MATLAB R2019a in the simulation. We assess performance using NMSE. We examined the NMSE performance of three channel estimation schemes—the classic OMP method, the OMP algorithm based on row-structured sparsity, and the scheme based on DS-OMP—to demonstrate the proposed approach’s superiority.

**Figure 5:**
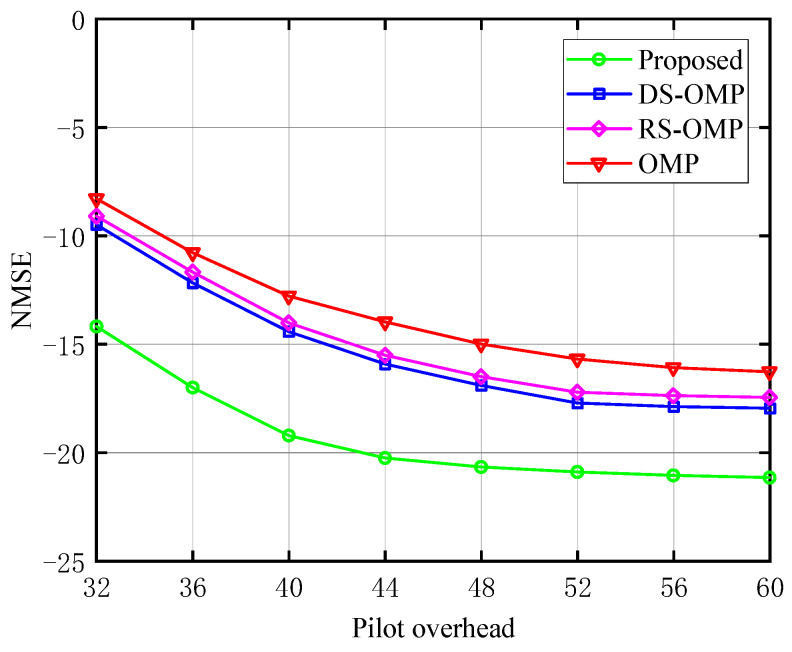
NMSE’s response to the pilot signal ($L_{c}=0$, compared with OMP algorithm [14], RS-OMP algorithm [13], and DS-OMP algorithm [15]).

**Figure 6:**
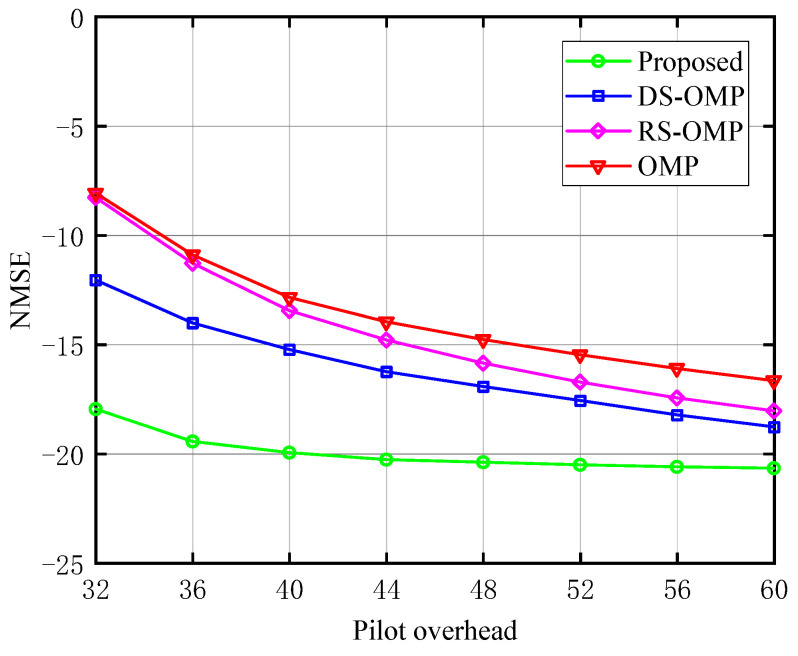
NMSE’s response to the pilot signal ($L_{c}=4$, compared with OMP algorithm [14], RS-OMP algorithm [13], and DS-OMP algorithm [15]).

**Figure 7:**
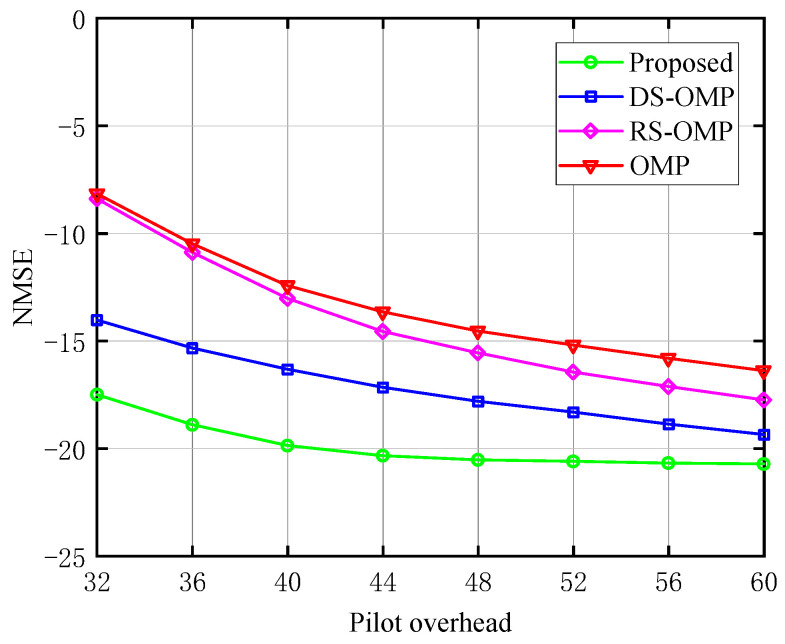
NMSE’s response to the pilot signal ($L_{c}=6$, compared with OMP algorithm [14], RS-OMP algorithm [13], and DS-OMP algorithm [15]).

The relationship between the NMSE and the pilot overhead of the BS-RIS-UE channel under various $L_{c}$ is depicted in Figure 5, Figure 6 and Figure 7, respectively. The work [15] discussed the performance of their algorithm in four different scenarios with $L_{c}$ of 0, 4, 6, and 8. We chose three of these ($L_{c}=0,4,6$) for comparison to fully demonstrate the performance advantages of our algorithm. The proposed channel estimation algorithm’s NMSE was contrasted with the conventional OMP approach, the OMP algorithm based on row-structured sparsity, and the algorithm based on DS-OMP. Under the same pilot overhead conditions, the proposed scheme outperforms the other three schemes in NMSE performance, and as the training pilots increase, our scheme consistently maintains significant performance advantages. Taking Figure 7 as an example, the estimation accuracy of the algorithm proposed in this article can approach $10^{-2}$ orders of magnitude when the pilot overhead is 44. The estimated accuracy of the other three algorithms is around $10^{-1.5}$ orders of magnitude lower. The proposed scheme is superior to the other three because it requires fewer pilots to perform more optimal channel estimation.

**Figure 8:**
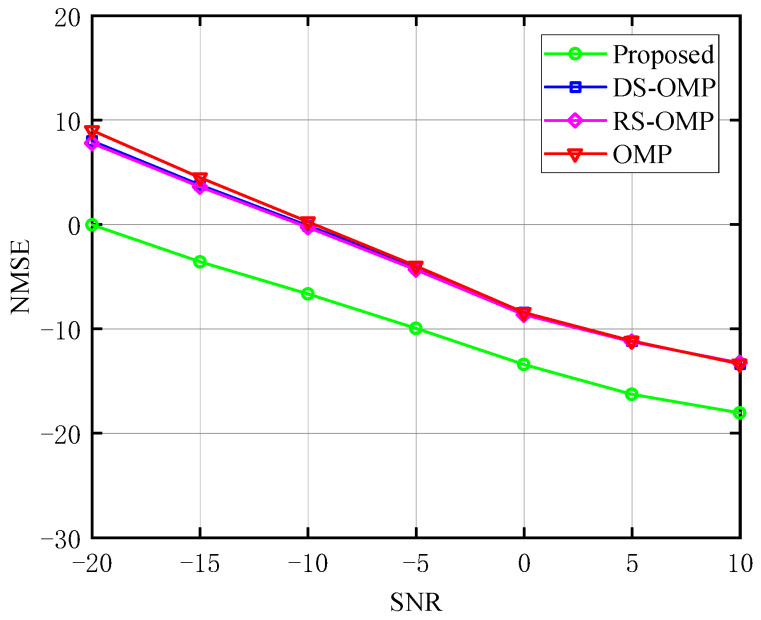
NMSE’s response to the SNR ($L_{c}=0$, compared with OMP algorithm [14], RS-OMP algorithm [13], and DS-OMP algorithm [15]).

**Figure 9:**
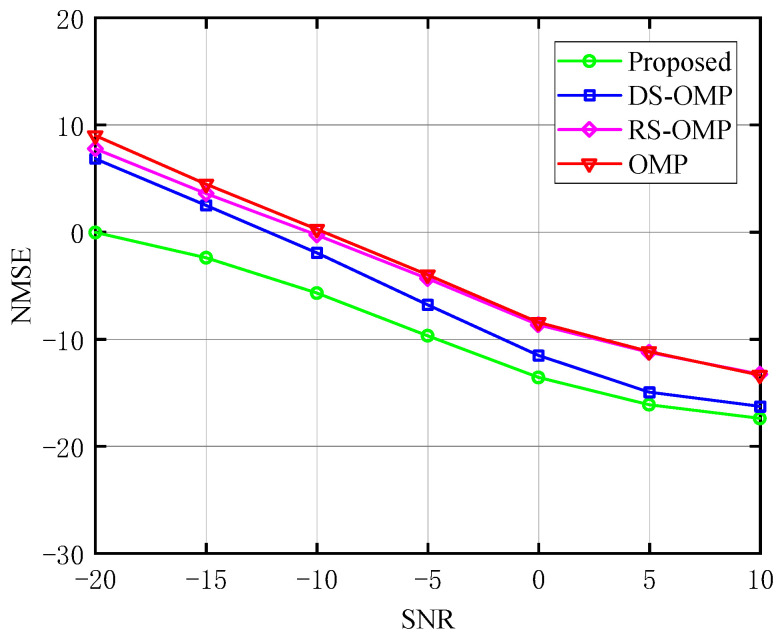
NMSE’s response to the SNR ($L_{c}=4$, compared with OMP algorithm [14], RS-OMP algorithm [13], and DS-OMP algorithm [15]).

**Figure 10:**
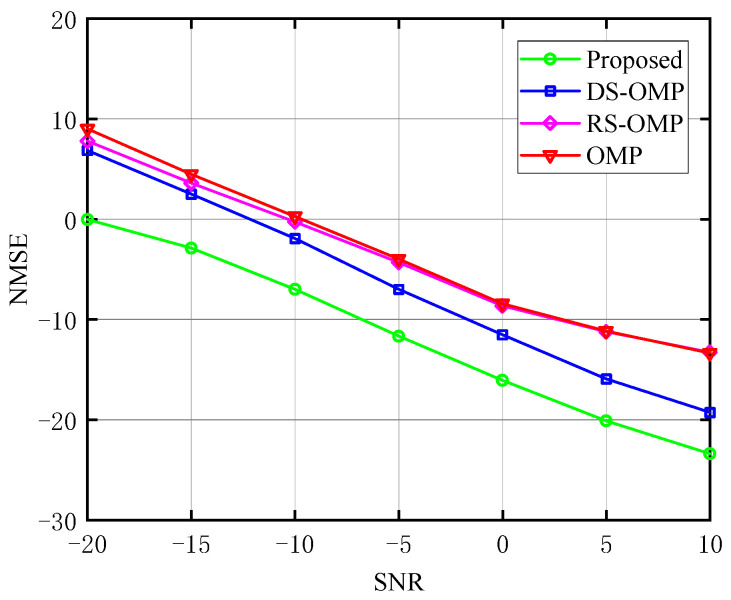
NMSE’s response to the SNR ($L_{c}=6$, compared with OMP algorithm [14], RS-OMP algorithm [13], and DS-OMP algorithm [15]).

The relationship between NMSE and SNR of the BS-RIS-UE channel under various $L_{c}$ is depicted in turn in Figure 8, Figure 9 and Figure 10. With the classic OMP method, the OMP algorithm based on row-structured sparsity, and the algorithm based on DS-OMP, we compared the NMSE of the proposed channel estimation scheme. Taking Figure 10 as an example, under the same *SNR*, the proposed method performs better in terms of NMSE than the other three schemes. It continuously retains a considerable performance advantage as the *SNR* rises, and our approach has an even greater advantage at low *SNR*. While the estimation accuracy of the other three schemes is roughly $10^{0.6}$ orders of magnitude when *SNR* = −20 dB, the approach proposed in this study has an estimation accuracy of $10^{0}$ orders of magnitude. The estimation accuracy of the algorithm proposed in this study can approach $10^{-1.8}$ orders of magnitude when *SNR* = 10 dB, whereas the estimation accuracy of the other three algorithms is only about $10^{-1.3}$ orders of magnitude, demonstrating the proposed algorithm’s superiority.

**Figure 11:**
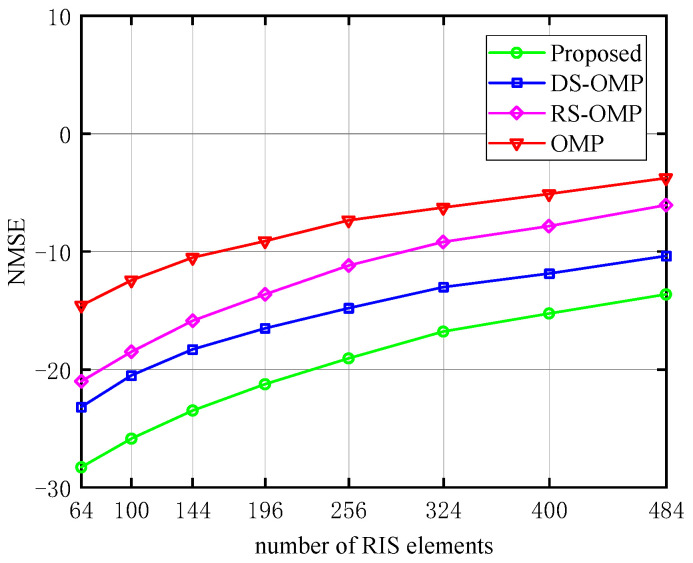
NMSE’s response to the number of RIS elements ($L_{c}=4$, compared with OMP algorithm [14], RS-OMP algorithm [13], and DS-OMP algorithm [15]).

**Figure 12:**
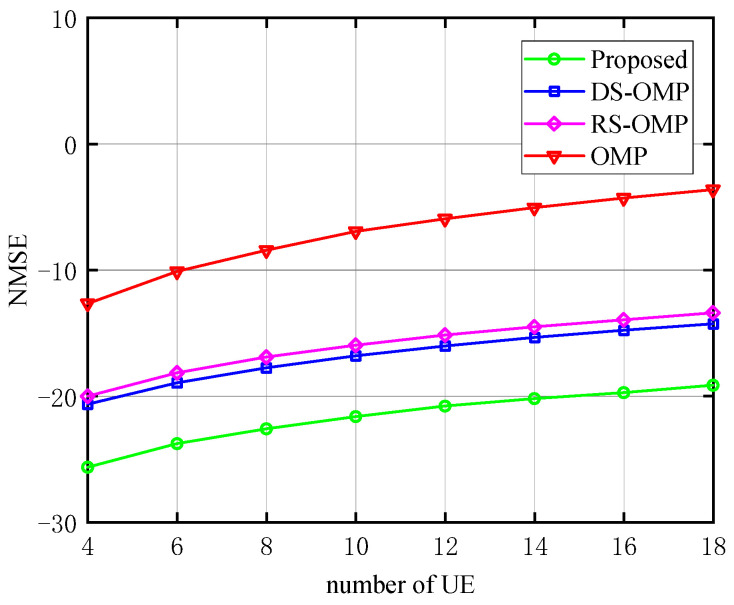
NMSE’s response to the number of UE ($L_{c}=4$, compared with OMP algorithm [14], RS-OMP algorithm [13], and DS-OMP algorithm [15]).

The relationship between NMSE and the number of RIS elements (*N*) and the number of UE (*K*) are depicted in turn in Figure 11 and Figure 12, respectively. Here, we chose one of the three $L_{c}$ above: $L_{c}$= 4. In Figure 11, under the same *N*, the proposed method performs better in terms of NMSE than the other three schemes. It continuously retains a considerable performance advantage as *N* rises. Due to a certain pilot cost, the estimation accuracy decreases with the increase of *N*. Therefore, the image shows an upward trend, and the same trend applies to the increase of *K* in Figure 12. While the estimation accuracy of the best of the other three algorithms is roughly $10^{-2.2}$ orders of magnitude when *N* = 64 in Figure 11, our proposed algorithm has an estimation accuracy of $10^{-2.8}$ orders of magnitude. The estimation accuracy of the proposed algorithm can approach $10^{-1.4}$ orders of magnitude when *N* = 484, whereas the estimation accuracy of the best of the other three algorithms is only about $10^{-1.0}$ orders of magnitude. In Figure 12, the estimation accuracy of the best of the other three algorithms is roughly $10^{-2.0}$ orders of magnitude when *K* = 4; our proposed algorithm has an estimation accuracy of $10^{-2.6}$ orders of magnitude. The estimation accuracy of the proposed algorithm can approach $10^{-2.0}$ orders of magnitude when *K* = 18, whereas the estimation accuracy of the best of the other three algorithms is only about $10^{-1.4}$ orders of magnitude, demonstrating the proposed algorithm’s superiority.

## 5. Conclusions

In this paper, we proposed an innovative two-phase channel estimation framework for an RIS-assisted multi-user uplink mmWave MIMO communication system. Within this framework, we proposed two channel estimation schemes based on the OMP algorithm to estimate the direct channels (BS-UE) and the cascaded channels (BS-RIS-UE), respectively. Specifically, the direct channels are estimated with an OMP-based LMMSE channel estimation algorithm that has higher estimation accuracy when considering low SNR. Then, an improved LMMSE algorithm based on DS-OMP is proposed for cascaded channels estimation using the double structured sparsity of angular domain cascaded channels in mmWave. The algorithm we propose is an iterative algorithm that can gradually improve the accuracy of channel estimation. In low SNR, using only the measurement matrix may not be able to accurately estimate the channel due to high measurement noise. However, by using the OMP algorithm to select paths with the maximum inner product, channel estimation can be performed on a smaller subset, thereby reducing the impact of noise. Moreover, LMMSE is utilized to process noise to further improve the quality of channel estimation. The simulation results show that compared with existing algorithms, our proposed algorithm has higher estimation accuracy under the same pilot overhead.

## Data Availability

Not applicable.
